# The Brain Resting-State Functional Connectivity Underlying Violence Proneness: Is It a Reliable Marker for Neurocriminology? A Systematic Review

**DOI:** 10.3390/bs9010011

**Published:** 2019-01-15

**Authors:** Ángel Romero-Martínez, Macarena González, Marisol Lila, Enrique Gracia, Luis Martí-Bonmatí, Ángel Alberich-Bayarri, Rebeca Maldonado-Puig, Amadeo Ten-Esteve, Luis Moya-Albiol

**Affiliations:** 1 Psychobiology Department, University of València, 46010 València, Spain; gonporma@alumni.uv.es (M.G.); Luis.Moya@uv.es (L.M.-A.); 2 Department of Social Psychology, University of Valencia, 46010 València, Spain; Marisol.Lila@uv.es (M.L.); Enrique.Gracia@uv.es (E.G.); 3 Biomedical Imaging Research Group (GIBI230), La Fe Health Research Institute, 46026 Valencia, Spain; marti_lui@gva.es (L.M.-B.); alberich_ang@gva.es (A.A.-B.); rebecagibi230@gmail.com (R.M.-P.); ten_ama@gva.es (A.T.-E.)

**Keywords:** anger state, brain, inmates, mental illness, resting functional connectivity, violence

## Abstract

**Introduction**: There is growing scientific interest in understanding the biological mechanisms affecting and/or underlying violent behaviors in order to develop effective treatment and prevention programs. In recent years, neuroscientific research has tried to demonstrate whether the intrinsic activity within the brain at rest in the absence of any external stimulation (resting-state functional connectivity; RSFC) could be employed as a reliable marker for several cognitive abilities and personality traits that are important in behavior regulation, particularly, proneness to violence. **Aims**: This review aims to highlight the association between the RSFC among specific brain structures and the predisposition to experiencing anger and/or responding to stressful and distressing situations with anger in several populations. **Methods**: The scientific literature was reviewed following the PRISMA quality criteria for reviews, using the following digital databases: PubMed, PsycINFO, Psicodoc, and Dialnet. **Results**: The identification of 181 abstracts and retrieval of 34 full texts led to the inclusion of 17 papers. The results described in our study offer a better understanding of the brain networks that might explain the tendency to experience anger. The majority of the studies highlighted that diminished RSFC between the prefrontal cortex and the amygdala might make people prone to reactive violence, but that it is also necessary to contemplate additional cortical (i.e., insula, gyrus [angular, supramarginal, temporal, fusiform, superior, and middle frontal], anterior and posterior cingulated cortex) and subcortical brain structures (i.e., hippocampus, cerebellum, ventral striatum, and nucleus centralis superior) in order to explain a phenomenon as complex as violence. Moreover, we also described the neural pathways that might underlie proactive violence and feelings of revenge, highlighting the RSFC between the OFC, ventral striatal, angular gyrus, mid-occipital cortex, and cerebellum. **Conclusions.** The results from this synthesis and critical analysis of RSFC findings in several populations offer guidelines for future research and for developing a more accurate model of proneness to violence, in order to create effective treatment and prevention programs.

## 1. Introduction

There is growing scientific interest in understanding the biological mechanisms affecting and/or underlying violent behaviors, in order to develop effective treatment and prevention programs [1,2,3]. In this regard, the relatively recent appearance of the field of neurocriminology represents an important advance in our understanding of these problems by applying the neuroscientific perspective to their study. In fact, neurocriminology aims to establish the neurobiological basis for crime and violence. Specifically, this neuroscientific subdiscipline has incorporated several tools and/or procedures, such as neuroimaging techniques, genetic markers, and hormonal measurements, among others, to predict these antisocial behaviors [3].

Neuroimaging techniques are non-invasive, and make it possible to visualize brain structures and functional connectivity in brain networks, thanks to their good spatial and functional resolution. Indeed, functional magnetic resonance imaging (fMRI) has offered insight into the functional synchrony between brain structures, that is, how the activation of several brain structures is temporally coordinated [4,5]. These techniques have usually relied on showing changes in the activation of different brain networks by analysing the blood oxygen level-dependent (BOLD) presented in these brain regions, which is especially sensitive to the increase in blood flow in the cerebral capillaries of the activated neuronal regions [6,7].

Studies using fMRI to assess brain networks have demonstrated that altered functional connectivity across distant brain regions might make individuals prone to violence [8,9,10,11,12,13,14,15]. In fact, most of the research in this field has highlighted that the alteration in the cerebral connectivity between the key nodes involved in emotional and cognitive behavioral regulation might explain this proneness to violence. Particularly, the inhibitory malfunction of the frontal lobe (i.e., prefrontal structures, frontal gyrus…) would lead to an overactivation of the limbic system (i.e., amygdala, hypothalamus, hippocampus…), which under certain stimuli might facilitate impulsive and/or reactive violence. In this regard, it has been suggested that the activation of frontal structures facilitates self-regulation and control over emotion-related behaviors by attenuating limbic responses to emotional stimuli and/or the context [8,9,10,11,12,13,14,15]. By contrast, several authors demonstrated that individuals characterized by predatory and instrumental violence (proactive) might present normal prefrontal cortex (PFC) functioning, and an increase in dorsolateral PFC activation has even been described during emotion processing tasks in these individuals [16,17,18,19]. Furthermore, studies have indicated that the reduction in amygdala activation during emotion processing might be characteristic of instrumental violence [19]. In this case, unimpaired or even higher frontal activation is related to the ability to control impulses and/or emotional processing, but also to certain alterations in empathic abilities. Unfortunately, the majority of the previously mentioned studies analyzed the functional activation of these brain structures in response to certain tasks (i.e., exposure to a stimulus, emotional induction tasks…), and so little is known about whether these brain structures and their connections in the absence of external stimulation might be employed as reliable markers of proneness to violence. 

In recent years, neuroscientific research has not only been interested in brain functioning during a task, but it has also focused on the intrinsic activity within the brain at rest without any external stimulation (resting-state functional connectivity; RSFC). In this direction, several studies have demonstrated the existence of resting intrinsic brain connectivity (networks) when an individual is awake and alert [20,21,22]. Synchronicity across a group of distant brain regions during resting periods has been called the default mode network (DMN; precuneus/posterior cingulate cortex, medial PFC and medial, lateral, and inferior parietal cortex). Although it has been suggested that the DMN is relatively deactivated during a demanding task [23,24,25], some authors have maintained that it might make an active contribution to cognitive processing [26,27], or even be important for cognitive abilities [28,29,30] or a correlate of certain personality traits [31,32]. With regard to the aforementioned, it should be mentioned that the activation of individual brain structures that encompass the DMN does not necessarily mean that the DMN is activated. In fact, it is necessary to consider how these brain structures establish and maintain networks with other brain structures outside the DMN during the resting state. Since evidence about whether the DMN or other brain networks might be important in violence proneness is less clear, it would be important to summarize the main results of this field of research.

In light of the above, this review aims to highlight the association between the RSFC among specific brain structures and the predisposition to experiencing anger and/or responding to stressful and distressing situations with anger. Specifically, we will focus on the following concepts related to violence: reactive violence (characterized by impulsivity, emotional drive, and lack of self-control); proactive violence (characterized by planning, high awareness of the purpose of this conduct, and low emotionality); trait anger (frequency of experiencing feelings of anger); and anger expression (frequency of externally and/or internally manifesting angry feelings). The present study will first describe the main findings on the association between RSFC and self-reported anger in non-violent normative individuals and in others with mental disorders. Then, we will describe the association between RSFC and changes in anger levels after laboratory tasks in non-violent and violent populations. Next, with the main purpose of analyzing whether these connections could be employed as reliable markers of violence proneness, we will analyze the RSFC in highly violent populations (i.e., young offenders, inmates, and soldiers) in comparison with non-violent individuals. Finally, considering the existing data, we will discuss the implications for clinical practice and further research.

## 2. Methods

### Search Strategy

A literature search on the existence of a relationship between brain RSFC and proneness to violence was carried out following the PRISMA quality criteria for reviews [33], using the following digital databases: PubMed, PsycINFO, Psicodoc, and Dialnet. The search terms were the following: Resting functional connectivity **or** resting fMRI **or** default mode network) **and** (violence **or** aggressive **or** anger).

**Figure 1:**
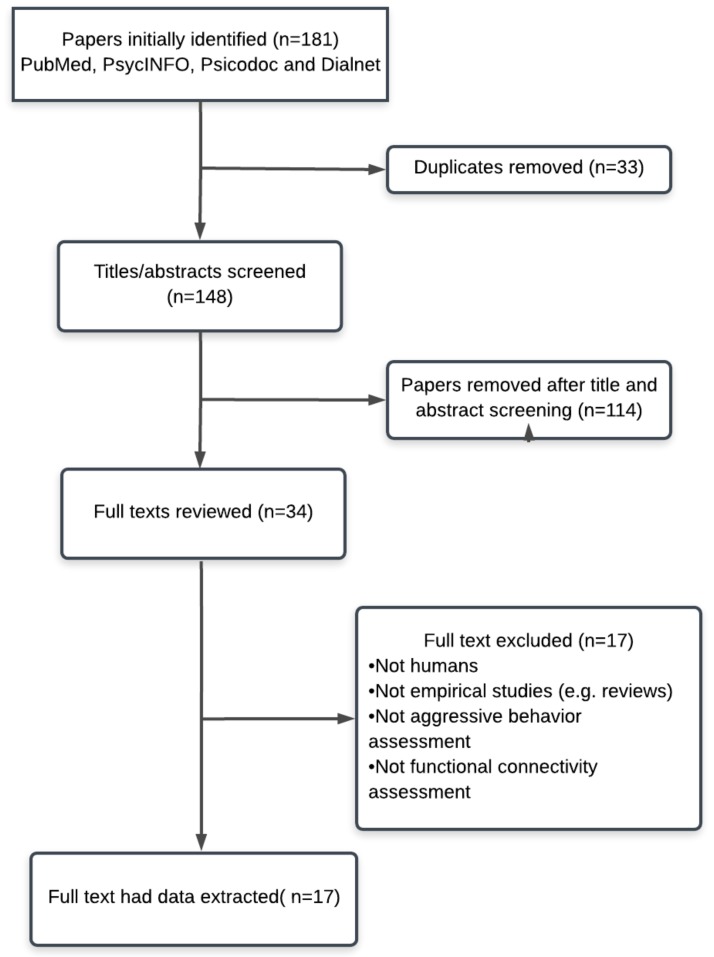
Flow chart of literature search with reasons for exclusion.

All of the papers that were selected for final inclusion met the following criteria: (a) they employed fMRI techniques to assess brain RSFC; (b) they were empirical studies; (c) they involved research with human subjects; and (d) they were written in English. Moreover, other criteria were not common to all of the papers, as they either: (a) examined anger through self-reports; (b) examined aggressive behavior through laboratory tasks; (c) examined RSFC in highly violent populations; (d) or assessed the relationship (i.e., correlational, regression analysis…) between RSFC and aggressive behavior as a trait and/or response to a laboratory task. Articles mentioning RSFC or aggressive behavior separately, but without examining the relationship between the two, were excluded. Moreover, studies were excluded if they did not directly examine anger or anger expression (i.e., externalizing behaviors, but not aggressive, criminal records…), or they did not explicitly mention that the participants were highly violent (PRISMA flow diagram; Figure 1).

Article selection was entrusted to two researchers. In cases of disagreement, a third member of the team helped them reach a consensus.

## 3. Results

After a systematic scientific literature search, we identified 181 abstracts, of which 34 texts were fully read because they seemed to present all of the inclusion criteria. Ultimately, only 17 of these papers were included in the review (Figure 1).

In all, 17 studies investigated the relationship between brain RSFC and several aspects of violence proneness (e.g., self-reported anger, changes in anger levels after a laboratory task-paradigm, or brain RSFC in a highly violent population). First, we will present the association between RSFC and self-reported anger in a non-violent normative population, followed by groups of non-violent patients with several mental disorders or brain damage (e.g., schizophrenia, bipolar disorder, attention deficit hyperactivity disorder (ADHD), and individuals with brain damage from traumatic brain injury). It should be noted that three studies mainly focused on the association between the RSFC and self-reported anger in carriers of specific alleles, and so we described the association by mentioning the specific allele subgroup. Third, we will describe whether brain RSFC predicts anger level changes after a laboratory method for inducing anger in a non-violent normative population, as well as in violent groups. Finally, we will describe the RSFC in different samples of highly violent populations (young offenders, inmates, and impulsive/violent soldiers). 

The main characteristics of the participants in each study are summarized in Table 1 (age, gender, education level, drug use, and handedness).

### 3.1. Normative Population (Self-Reported Aggression)

One study investigated whether RSFC was associated with trait anger in normative children (both genders). Its authors concluded that low functional connectivity between the bilateral amygdala and ventromedial prefrontal cortex (vmPFC) was related to higher levels of trait aggression (assessed by the Child Behavior Checklist), but this relationship did not remain significant after controlling for family income and maternal education [34]. 

Another study with young adults added to the previous results by studying whether the amygdala also presented connections with other prefrontal cortex (PFC) structures that facilitate proneness to experiencing anger feelings. In fact, Fulwiler, King, and Zhang [35] studied whether the amygdala’s functional connectivity with other PFC structures predicted trait aggression in young men. These authors demonstrated that, in men, low RSFC between the amygdala (bilateral) and left orbitofrontal cortex (OFC) was associated with high trait anger (measured by the State-Trait Anger Expression Inventory 2, STAXI-2), especially for the right amygdala and left middle orbitofrontal cortex (mOFC). Conversely, the high functional connectivity between these brain structures was related to high anger control-out; in other words, the higher the association between these two brain structures, the greater the effort to control the expression of anger toward others (persons or objects) [35]. 

Finally, the interconnections between the amygdala and the PFC have not only been studied as facilitators of anger. Abram, Wisner, Grazioplene, Krueger, MacDonald, and DeYoung [36] conducted a study with a relatively larger sample of healthy young adults (of both genders). They assessed whether RSFC was associated with several facets of callous aggression (assessed by externalizing spectrum inventory), such as relational, physical, and destructive aggression. In this regard, they concluded that there was a positive association between the anterior insula, ventral striatum (VStr), and anterior cingulate cortex (ACC), connectivity and physical aggression. However, they also found a negative association between anterior insula and OFC connectivity and physical aggression and destructive aggression.

### 3.2. Self-Reported Aggression Mediated by Genetic Markers

Two studies included genetic markers as mediators of the relationship between RSFC and trait aggression in a sample of young males. One of these studies concluded that Monoamine oxidase A-L (MAOA-L) carriers (in comparison with MAOA-H carriers) showed that a higher RSFC between the ventromedial prefrontal cortex (vmPFC) and the right angular gyrus (AG), posterior cingulate cortex (PCC), and dorsomedial prefrontal cortex, was related to higher aggression traits. Nevertheless, when the RSFC between the vmPFC and bilateral supramarginal gyrus was high, the aggressive traits were low [37]. The other study revealed that in participants with antisocial personality traits with the MAOA-L variant, high RSFC between the ventral striatal and angular gyrus was related to high proactive aggression [38].

### 3.3. Mental Disorders (Self-Reported Aggression)

In order to avoid biased results, it is important to consider whether and how these variables are related in a normative population and in people with mental disorders, in order to be able to generalize the conclusions to the entire population. For example, in schizophrenic patients, low functional connectivity (bilaterally) between the amygdala and the ventromedial prefrontal cortex (vmPFC) was related to a higher total score on the Buss–Perry Aggression Questionnaire (BPAQ), a life history of aggression, and the total number of arrests. However, the functional connectivity between the bilateral amygdala and the bilateral orbitofrontal cortex (OFC), supragenual cingulate, subgenual cingulate, or dorsolateral prefrontal cortex (DLPFC) regions was unrelated to the aforementioned aggressive behavior scales in schizophrenic patients. These results remained significant even after controlling for the patients’ diagnosis (schizophrenia and schizoaffective disorder), age, and neuroleptic medication dosage [39]. 

Nevertheless, not all of the studies considered the connections between the amygdala and the PFC. Specifically in bipolar disorder, the assessment of functional connectivity and the BPAQ total score in a group of women with borderline personality disorder demonstrated that low functional connectivity between the serotonergic nucleus centralis superior (NCS) and the frontopolar cortex (FPC) was associated with higher BPAQ total scores in unmedicated bipolar patients. However, the RSFC between these brain structures was unrelated to a control group [40].

In ADHD patients, a study demonstrated that higher connectivity between the right hippocampus, the parietal lobe (supramarginal and angular gyrus), and the frontal lobe (superior and middle frontal gyrus) in carriers of the AG or GG variant of rs8079626 within the BAIAP2 gene (brain-specific angiogenesis inhibitor) was related to higher anger expression-out (or an unmanageable tendency to manifest anger externally) in ADHD patients [41].

In patients with brain damage after traumatic brain injury (TBI), the reduction in the functional connectivity between the left orbitofrontal cortex (OFC) and the left angular region was related to higher scores on the BPAQ physical aggression subscale in male veterans with TBI. Moreover, high RSFC between the right OFC and the right cerebellum and right angular gyrus was related to high scores on the Displaced Aggression Questionnaire (DAQ) Revenge Planning scale in this group. Conversely, connectivity between the right OFC and the right mid occipital cortex was negatively correlated with the scores on the DAQ Revenge Planning scale. Finally, it should be noted that the frontal cortex (FC) of these brain regions was unrelated to BPAQ and/or DAQ scores in female veterans with TBI [42].

Although a study that analyzed the RSFC of a group of retired athletes with a history of multiple concussions failed to find a significant relationship between the RSFC of the bilateral anterior temporal lobe (ATL) and the bilateral medial orbitofrontal cortex (mOFC) and the PAI aggression subscale [43], a recent study demonstrated that in a group of adults with TBI, high RSFC between the right hippocampus and midcingulate cortex was associated with elevated BPAQ scores. However, in a control group, higher self-reported aggression was associated with low RSFC between the right hippocampus and midcingulate cortex, as well as between the right hippocampus and mPFC [44]. Finally, it should be noted that the majority of the studies that analyzed the associations between brain RSFC and violence-related concepts found significant results related to the BPAQ questionnaire [39,40,42,44], but they failed to find these associations on other self-reports such as the PAI. In this regard, a possible explanation for this difference would be that the BPAQ is a self-report to analyze violence-related concepts, unlike the PAI (e.g. total score consists of the average of approximately 29 items), which is a broader personality self-report with only a few questions about violence.

### 3.4. Laboratory Assessment of Aggression

The assessment of anger expression under controlled laboratory conditions offers an interesting alternative to self-reports, although these methods also have some limitations, such as artificial conditions, social biases, the simplification of violence measurement, etc. However, they offer complementary information to self-report assessments of violence-related concepts. 

#### 3.4.1. Non-Violent Groups

A group of men were submitted to a modified version of the Ultimatum Game, which was employed to induce anger. During this laboratory task, participants have to split a specific amount of money with another player by using negotiation strategies. However, the other player breaks the rules of the game and confronts his opponent directly and insults him/her. Participants experienced an increase in the right amygdala and right inferior frontal gyrus (IFG) functional connectivity after this laboratory task, which, in turn, was associated with higher trait anger scores. Moreover, it should also be noted that individuals with high pre-task functional connectivity in the right amygdala had low anger levels during the task [45].

Another study analyzed whether being exposed to a modified version of a reaction time task (Social Threat Aggression Paradigm) to promote anger induction would produce changes in the RSFC among different brain structures in a sample of healthy young women. On this task, participants had to punish an opponent if he/she (loser) presented longer reaction times than the participant (winner). The punishment consisted of an aversive tone whose intensity was manipulated by the winner. The authors concluded that although the resting functional connectivity (before the laboratory task) among the basolateral amygdala (BLA), the medial orbitofrontal cortex (mOFC), and the lateral orbitofrontal cortex (OFC) (bilaterally) was unrelated to the anger levels in the Social Threat Aggression Paradigm (a laboratory task), participants who presented higher increases in BLA–mOFC connectivity after the task showed a less aggressive response to this task [46]. Moreover, high baseline RSFC between the BLA and the left superior temporal gyrus (STG) was correlated with high aggression levels on the laboratory task. By contrast, high basal connectivity between the BLA and the superior parietal lobule (SPL) was associated with lower aggression [46].

#### 3.4.2. Violent Group

Two groups of men (reactive violent offenders and non-violent offenders) participated in a laboratory task to promote different emotional states (they had to pay attention to several audiotaped angry, happy, and/or neutral stories). This is an adapted version of the Anger Articulated Thoughts during Simulated Situations (ATSS) paradigm for fMRI. Authors of the study concluded that before the emotional task, reactive violent offenders showed a heightened RSFC between the left medial PFC (mPFC) and the left amygdala, as well as a diminished pre-task RSFC between the left amygdala and the uncus/amygdala and posterior insula. After the emotion task, violent offenders showed a significant decrease in the RSFC between the left mPFC and the left amygdala, but non-offender controls experienced an increase in RSFC. Furthermore, an increase was found in the RSFC between the left amygdala and the right posterior insula and right superior temporal gyrus in violent offenders after the task, but the control group showed a decreased RSFC between the left amygdala and the right posterior insula and right superior temporal gyrus, as well as the left uncus/amygdala [47].

## 4. Violent Populations

Finally, in order to complete the previously mentioned results, it is important to analyze whether individuals with a proneness to expressing anger present alterations in the RSFC among the brain structures that have been identified as responsible (at least in part) for violence-related concepts. First, violent juvenile offenders showed significantly lower resting regional connectivity between the following brain structures and their adjacent structures: the right caudate, right medial prefrontal cortex, and left precuneus. However, significantly higher RSFC was also found between the right supramarginal gyrus and its adjacent brain structures, compared to the non-violent control group [48].

One study compared the RSFC of a group of violent inmates to that of a non-violent group, and they concluded that the violent offender group showed an increase in the RSFC between the left amygdala and the right cerebellar hemisphere. Furthermore, these violent offenders also presented an increase in the RSFC with the dorsolateral prefrontal cortex (DLPFC) (bilateral). Nonetheless, inmates showed a decrease in RSFC between the left/right cerebellar hemisphere and the left/right orbitofrontal cortex (OFC), as well as between the vermis and the left OFC [49].

Varkevisser, Gladwin, Heesink, van Honk, and Geuze [50] compared the RSFC of a group of aggressive and impulsive soldiers to a non-violent group. Although no significant group differences in functional connectivity were found between the orbitofrontal cortex and basolateral amygdala, significant patterns of connectivity were found in impulsive and aggressive soldiers. First, these authors concluded that the left dorsolateral prefrontal cortex presents a negative association with the (bilateral) basolateral amygdala. Nevertheless, they found a positive association between the left centromedial (CeM) amygdala and a region spanning the left fusiform gyrus and lingual gyrus, as well as between the left anterior cingulate cortex (ACC) and a region spanning the left cuneus, calcarine cortex, and superior occipital cortex. Furthermore, they also found a positive association between the right ACC and a region spanning the left cuneus, calcarine cortex, superior occipital cortex, and precuneus. Finally, they demonstrated a positive relationship between the left anterior insular cortex (AIC) and the right temporal pole. However, in the control group, the associations among these brain structures presented the opposite connectivity pattern.

## 5. Discussion

The results described here offer a better understanding of RSFC that might explain proneness to violence, particularly the neural pathways that underlie key variables of violence, such as reactive and proactive violence, trait anger, and anger expression and/or control. It should be noted that only one study failed to find a significant association between the RSFC of the bilateral anterior temporal lobe and mOFC and the anger trait; the other 16 manuscripts did find a significant association between these variables. Even though several studies highlighted that the diminished RSFC between the PFC and the amygdala increased proneness to reactive violence, we also summarized other brain networks that include additional cortical (i.e., insula, gyrus (angular, supramarginal, temporal, fusiform, superior and middle frontal), ACC, and PCC) and subcortical brain structures (i.e., hippocampus, cerebellum, ventral striatum, and nucleus centralis superior) that might facilitate the onset of this type of violence. Moreover, we also described the neural pathways that might explain proactive violence and feelings of revenge, which are focused on the RSFC between the OFC, ventral striatal, angular gyrus, mid-occipital cortex, and cerebellum (Table 2).

Initially, we concluded that the diminished RSFC between frontal structures such as the PFC (OFC and vmPFC) and frontopolar cortex (Brodmann area 10; PFC), limbic structures such as the amygdala and the anterior insula, parietal cortex regions such as the supramarginal gyrus (Brodmann area 40; parietal lobe), the angular region (posterior to the supramarginal gyrus), and the nucleus centralis superior (median raphe nucleus; brainstem) was related to a high predisposition to experiencing anger and/or responding to stressful and distressing situations with anger (high anger expression-out and low control-out) in a normative population and in participants with mental disorders. 

As previously stated, it appears that prefrontal structures tend to maintain inhibitory projections to other brain structures involved in emotional reactivity, such as the amygdala and/or the insula, and so the failure to regulate this reactivity might lead to high irritability or hostile reactions [8,9,10,11,12,13,14,15]. Although these studies did not analyze the RSFC between these brain structures, all of the manuscripts that were included in this systematic review found a diminished RSFC connectivity between the PFC and amygdala, which has been associated with proneness to experiencing feelings of anger and difficulties in controlling anger expression [34,35,39,46,47,50]. In fact, alterations in the dorsal and ventral PFC, amygdala, and angular gyrus have commonly been associated with rule-breaking behaviors, alterations in moral judgement and reasoning, and emotion regulation [8]. In this regard, we reinforced the hypothesis that diminished RSFC between the frontal and limbic systems might be characteristic of violent populations, since one of the studies that was included in this review concluded that a group of violent and impulsive soldiers presented a lower RSFC between the left dorsolateral PFC and the basolateral amygdala (bilateral) than a non-violent group [47,50]. Conversely, research conducted with normative young adults demonstrated that individuals with higher RSFC between the mOFC and the basolateral amygdala showed less aggressive strategies on an anger induction laboratory task [46]. Moreover, another study stated that reactive violent offenders presented a diminished RSFC between the left mOFC and left amygdala, but an increase in paralimbic RSFC after an emotion induction task [47].

Although it appears that the diminished RSFC between the PFC and amygdala might be employed as a useful marker for proneness to violence, other brain networks should be considered in order to offer a broader understanding of the complex phenomenon of violence. Even though other limbic structures and their projections are involved in proneness to violence, we can conclude that high RSFC between the amygdala and the inferior frontal gyrus and left superior temporal gyrus was associated with high anger traits in several populations (normative and with mental disorders). Furthermore, the amygdala and the ACC maintained high RSFC with the fusiform and lingual gyrus, cuneus and precuneus calcarine cortex, and superior occipital cortex in impulsive and aggressive individuals. Recently, a research group demonstrated that violent individuals with schizophrenia presented hyperactivation of the ACC when perceiving negative images [51], especially in highly impulsive schizophrenic individuals [52]. This result coincides with the hypothesis that a large percentage of violent offenders present an attentional bias toward negative stimulus and/or a hostile attribution bias [53]. Moreover, other brain networks composed of the inferior frontal and temporal gyrus, ACC, and anterior insula are involved in voluntarily and actively sharing the emotional experience of other individuals (through intentional empathic processes), which is extremely important in behavioral regulation [8]. Thus, we sustain that this specific brain network facilitates the onset of violence due to attributing negative and hostile intentions to others, which is congruent with the scientific literature in this field.

Regarding other limbic structures, the hippocampus and the anterior insula maintain anger expression facilitation projections with the supramarginal and angular gyrus, the superior and middle frontal gyrus, the ventral striatum, and the ACC. In this regard, the middle frontal gyrus plays an important role in hostile cognitive bias and angry rumination (or how often an individual tends to re-experience negative feelings) [54,55]. We also hypothesized that the heightened connectivity between the left/right orbitofrontal cortex (OFC) in violent inmates [49] may play a role in angry rumination. Furthermore, it was recently demonstrated that brainstem alterations (except its volume) seem to partly explain high irritability and proneness to react violently in males with autism [12,13]. Moreover, in two groups of violent inmates, the authors concluded that the PFC (mPFC and OFC) also maintained lower connectivity with adjacent areas and the cerebellar vermis, compared to non-violent males [41,42], with the cerebellum joined to the basal ganglia, and the supplementary motor area being important in impulse control [9]. Therefore, we might summarize that these RSFC patterns in a cognitive model where high anger traits that were linked to empathic alterations (i.e., perspective-taking disruptions, emotion-decoding deficits…), hostile cognitive biases, and heightened anger rumination might lower the threshold for reacting with violence in ambiguous contexts, physically facilitating reactive violence.

With regard to proactive violence, one study that was included in our review used self-reports to analyze the RSFC underlying this kind of violence. However, the authors did not compare reactive and proactive violence, and so it may be difficult to establish in this review whether there are differentiated neural pathways to each kind of violence [56]. Kolla, Dunlop, Meyer, and Downar [38] concluded that high RSFC between the ventral striatal and the angular gyrus was associated with high proactive violence strategies. Curiously, psychopaths (who usually but not always employ proactive violence) have been found to present structural abnormalities in the striatum—particularly increased volume—compared to non-psychopaths [57]. Moreover, it has been suggested that the angular gyrus belongs to the brain networks underlying moral reasoning [8]. Thus, these results offer information about which neural pathways underlie this kind of violence, which is defined as predatory, and is often characterized by premeditation and being cold-blooded [58]. Extending these findings, another study [42] revealed that revenge feelings were positively correlated with the RSFC between the OFC and the cerebellum, angular gyrus, and midoccipital cortex. Furthermore, recent research highlighted that psychopathic traits, as well as the risk of reoffending, maintained a positive relationship with the grey matter volume of the cerebellum [9]. If we try to explain why these neural pathways explain revenge feelings, we can assume that these neural pathways are associated with several processes that are important for revenge, such as the anticipation of consequences of violent behavior (good or bad) and reimagining different contexts to consume the desire for revenge [58]. 

**Figure 2:**
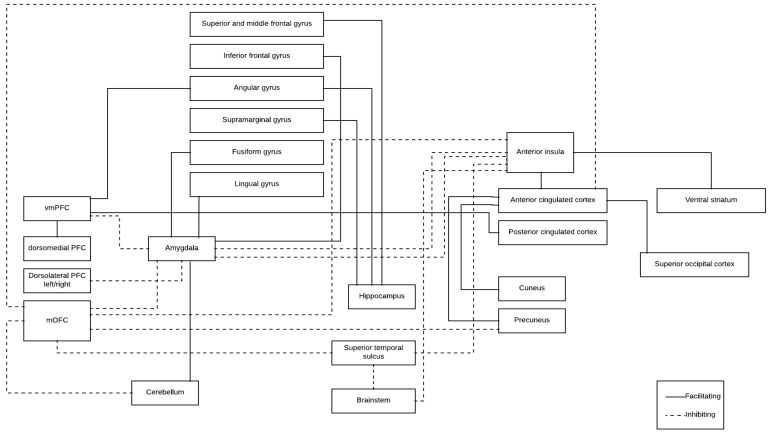
Proposed model of facilitating and inhibiting brain networks for reactive violence proneness.

Finally, one way to prevent these antisocial behaviors is to promote empathic abilities in violent populations through various psychological interventions. Thus, it has been hypothesized that there is an overlap between these brain regions that underlie violence and empathy, but the pattern of functional connectivity underlying each of them is inverse [57]. Regarding RSFC, a recent study demonstrated that individuals (men and women) with higher empathic abilities tend to present greater RSFC between the mPFC and the dorsal ACC, the precuneus, and the left superior temporal sulcus [58]. Conversely, low levels of empathy have been related to lower RSFC between the mPFC and ACC [30]. Moreover, it has been stated that high affective empathy tends to be related to stronger RSFC between the ventral anterior insula, OFC, amygdala, and perigenual anterior cingulated, and that high cognitive empathy is related to greater RSFC between the brainstem, superior temporal sulcus, and ventral anterior insula [59]. This statement is congruent with the assertion that there is a certain overlap between the brain structures that underlie violence and empathy, but they present inverse RSFC patterns. Thus, we might conclude that the brain RSFC in empathic abilities is more harmonious (i.e., positive RSFC between the PFC and the limbic system), than the pattern for violence (i.e., negative RSFC between PFC and the limbic system), which describes an imbalance in the brain functioning of individuals who tend to present proneness to violence. Based on the summarized results of our review and the brain RSFC underlying empathy, we proposed the facilitating and inhibiting brain networks for reactive violence described in Figure 2.

Three important limitations of the studies carried out are the lack of a homogeneous population and their limited sample size. In fact, there is great diversity in the relevant variables, and not all of the studies reported or controlled the potential confounding effects of variables such as handedness, drug use, educational level, economic level, ethnicity, and psychopathology, among others. Furthermore, only a few studies included women, and so most of the research was conducted with males. Although small sample sizes kept us from properly estimating the differences between groups, most of the studies applied Bonferroni corrections for multiple comparisons. Moreover, they employed different anger trait questionnaires that measure different facets of anger (trait, physical, or verbal tendency to express anger, anger expression in general, proactive violence…). Lastly, several of the articles that were included in our review based their conclusions on seed-based findings that are necessarily limited to a priori regions. In this regard, the absence of a finding in another region is very different from a study that may have used ICA. Hence, it is difficult to obtain unanimous conclusions about the association between RSFC and anger.

In summary, the present review study confirmed that reduced frontal and limbic connectivity is a good correlate for several variables, such as trait anger and anger expression or control, which are important in violence proneness. Nevertheless, in order to offer a broader understanding of violence proneness, we need to contemplate other resting-state brain networks, including cortical regions (i.e., gyrus, parietal, temporal, ACC…) and subcortical structures (i.e., hippocampus, insula, brain stem, cerebellum…), in addition to the PFC and amygdala. Therefore, based on the studies summarized in this manuscript, along with those that analyzed the RSFC underlying empathic processes, we proposed a model to explain anger proneness to reactive violence (Figure 2). In sum, our review offered more insight into the importance of studying the brain RSFC underlying several important processes for violence proneness. It also reinforced the need to carry out further studies that analyze the importance of the RSFC using larger sample sizes, contemplating several populations, and employing a common anger assessment. Moreover, it would be necessary to check whether these RSFC could be considered temporally stable (as a ‘trait’), or whether they might change after interventions focused on promoting behavioral regulation and cognitive improvements. In fact, several studies demonstrated that after a focused intervention to promote cognitive and empathic changes in groups of intimate partner violence (IPV) perpetrators, these individuals improved specific cognitive and empathic abilities [60]. Thus, this research allows us to detect whether these changes correspond to specific brain network changes. Therefore, caution should be used in interpreting these results, in order to develop effective intervention programs to reduce proneness to violence.

## Figures and Tables

**Table 1:** Main sociodemographic characteristics and details about the participants in each study and the assessment methods used. ADHD: attention deficit hyperactivity disorder, fMRI: functional magnetic resonance imaging, TBI: traumatic brain injury.

Authors	Sample Characteristics	Age	Gender	Education	Drug Use	Handedness (Right/Left)	Violent Behavior Assessment	Methods of Analysis
Park et al. (2018) [34]	Healthy young children (n = 79)	6.06 ± 0.96	49% ♂ 51% ♀	-	-	-	Child Behavior Checklist	Seed-based
Fulwiler et al. (2012) [35]	Healthy males (n = 16)	34 ± 14.42	♂	-	No current drug use	Right-handed	Spielberger State–Trait Anger Expression Inventory-2	ROI
Abram et al. (2015) [36]	Psychiatry healthy sample (n = 244)	26	50% ♂ 50% ♀	-	No current drug use	Right-handed	Externalizing Spectrum Inventory, brief form	ICA
Klasen et al. (2018) [37]	Healthy young adults (n = 83)	23.8 ± 3.6	♂	-	-	Right-handed	Buss–Perry Aggression Questionnaire	ROI
Kolla et al. (2018) [38]	Antisocial personality disorder subjects (n = 21) Controls (n = 19)	36.2 ± 8.7 34.2 ± 7.7	♂	-	No current drug use	-	Buss–Perry Aggression Questionnaire Reactive–Proactive Aggression Questionnaire	ROI
Hoptman et al. (2010) [39]	Patients with schizophrenia or schizoaffective disorder (n = 25) Controls (n = 21)	36.7 ± 10.5 40.4 ± 10.8	88% ♂ 12% ♀ 76% ♂ 24% ♀	12.3 ± 2.1 (years) 15.5 ± 3.0 (years)	CPZ equivalents	-	Buss Perry Aggression Questionnaire Life history of aggression Number total of arrests	ROI
Wagner et al. (2018) [40]	Unmedicated female patients with BPD (n = 33) Controls (n = 33)	26.7 ± 6.4 26.4 ± 6.2	♀	12.1 ± 1.6 (years) 11.8 ± 1.5 (years)	No current drug use	.	Buss–Perry Aggression Questionnaire	ROI
Hasler et al. (2017) [41]	ADHD (n = 30) Controls (n = 15)	38.7 ± 9.9 32.2 ± 5.5	70% ♂ 30% ♀ 26% ♂ 73% ♀	-	48h free of methylphenidate before fMRI	Right handed	Spielberger State–Trait Anger Expression Inventory-2	CO_2_ challenge regressor
McGlade et al. (2015) [42]	Veterans males with TBI (n = 24) Veterans females with TBI (n = 17)	37.75 ± 9.59 40.0 ±11.15	♂ ♀	14.33 ± 2.10 (years) 15.06 ± 2.51 (years)	-	-	Buss–Perry Aggression QuestionnaireDisplaced Aggression Questionnaire	Seed-based
Goswami et al. (2016) [43]	Retired athletes with a history of multiple concussions (n = 19); Controls (n = 17)	50 ± 12 46 ± 10	♂	17 ± 1.8 16 ± 1.9	No current drug use	-	Personality Assessment Inventory (aggression scale)	Seed-based
Dailey et al. (2018) [44]	Adults with TBI (n = 17) Healthy controls (n = 17)	21.86 ± 2.79 23.88 ± 3.26	26% ♂ 73% ♀ 29% ♂ 71% ♀	-	-	-	Buss–Perry Aggression Questionnaire	ROI
Gilam et al. (2017) [45]	Soldiers (n = 60)	18.62 ± 0.88	♂	> secondary education	No current drug use	Right-handed	Spielberger State–Trait Anger Expression Inventory Geneva Emotion Wheel	Brain functional parcellation
Buades-Rotger et al. (2018) [46]	Healthy young women (n = 39)	23.22 ± 3.2	♀	-	No current drug use	Right-handed	Social Threat Aggression Paradigm	ROI
Siep et al. (2018) [47]	Violent offenders (n = 18) Non-offender controls (n = 18)	35.17 ± 7.12 37.06 ± 15.24	♂		Current alcohol use No current drug use	-	-	Seed-based
Chen et al. (2015) [48]	Young violent offenders (n = 30) Controls (n = 29)	16.06 ± 0.7 16.06 ± 0.4	♂	7.76 ± 2.2 10.06 ± 0.0	-	Right-handed	-	ROI
Leutgeb et al. (2016) [49]	Violent inmates of maximum security prison (n = 31) Controls (n = 30)	36.8 ±12.0 35.1 ± 9.0	♂	11.3 ± 1.7 (years) 11.6 ± 1.0 (years)	Non-medicated	Right-handed	-	Seed-based
Varkevisser et al. (2017) [50]	Impulsive and violent soldiers (n = 28) Controls (n = 30)	36.54 ± 6.27 34.53 ± 7.59	♂	67,9% middle 53,3% middle	-	-	Interviews and criminal records	ROI

**Table 2:** Resting functional connectivity and its role in anger pronenes. mOFC: medial orbitofrontal cortex, OFC: orbitofrontal cortex, PFC: prefrontal cortex, vmPFC: ventromedial prefrontal cortex.

Brain Structure (From)	Brain Structure (To)	Functional Connectivity	Aggression Assessment
Trait aggression			
Amygdala (bilateral)	Left mOFC	⬇	⬆ Trait aggression
	vmPFC	⬇	⬆ Trait aggression
Nucleus centralis superior (median raphe nucleus)	Frontopolar cortex (Brodmann area 10)	⬇	⬆ Trait aggression
vmPFC	Bilateral supramarginal gyrus	⬇	⬆ Trait aggression
Amygdala (right)	Inferior frontal gyrus (right)	⬆	⬆ Trait aggression
vmPFC	Angular gyrus (right) Posterior cingulated cortex Dorsomedial PFC	⬆	⬆ Trait aggression
Anger expression			
Amygdala (bilateral)	Left mOFC	⬇	⬇ Anger control-out
Hippocampus (right)	Parietal (supramarginal and angular gyrus) Frontal lobe (superior and middle frontal gyrus)	⬆	⬆ Anger expression-out
Proactive aggression			
Ventral striatal	Angular gyrus	⬆	⬆ Proactive aggression
Physical aggression			
OFC (left)	Left angular region	⬇	⬆ Physical aggression
Anterior insula	OFC	⬇	⬆ Physical aggression ⬆ Destructive aggression
Anterior insula	Ventral striatumAnterior cingulate cortex	⬆	⬆ Physical aggression
Revenge feelings			
Right OFC	Right cerebellum Right angular gyrus	⬆	⬆ Revenge feelings
Right OFC	Right midoccipital cortex	⬆	⬇ Revenge feelings
Laboratory task			
Amygdala basolateral	Left superior temporal gyrus	⬆	⬆ Aggressive strategies (laboratory task)
	mOFC Superior parietal lobule	⬇	⬆ Aggressive strategies (laboratory task)
Left mOFC	Left amygdala	⬆	Before emotional induction task (violent offenders)
Left amygdala	Left uncus/amygdala Posterior insula	⬇	Before emotional induction task (violent offenders)
Left mOFC	Left amygdala	⬇	After emotional induction task (violent offenders)
Left amygdala	Right posterior insula Right superior temporal gyrus	⬆	After emotional induction task (violent offenders)
Violent populations (no-self reported)			
Caudate nucleus (right) mPFC (right) Precuneus (left)	Adjacent structures	⬇	Young violent offenders
Supramarginal gyrus (right)	Adjacent structures	⬆	Young violent offenders
Right cerebellar hemisphere	Left amygdala	⬆	Violent inmates
Bilateral cerebellar hemisphere	Bilateral OFC	⬇	Violent inmates
Cerebellar vermis	Left OFC	⬇	Violent inmates
Left dorsolateral PFC	Right dorsolateral PFC	⬆	Violent inmates
Amygdala basolateral (bilateral)	Left dorsolateral PFC	⬇	Impulsive and aggressive group
Left centromedial amygdala	Left fusiform gyrus Lingual gyrus	⬆	Impulsive and aggressive group
Left anterior cingulate cortex	Left cuneus Left calcarine cortex Left superior occipital cortex	⬆	Impulsive and aggressive soldiers
Right anterior cingulated cortex	Left cuneus Left precuneus Left calcarine cortex Left superior occipital cortex	⬆	Impulsive and aggressive soldiers
